# Cushing's Storm: A Case of Paraneoplastic Cushing's Syndrome-Induced Seizures Treated With Continuous Etomidate Infusion

**DOI:** 10.7759/cureus.69193

**Published:** 2024-09-11

**Authors:** Christopher B Marsalisi, Yixin Zhang, John M Sousou, Loruanma Lam, Landen S Burstiner, Jason Ferreira, Nimeh Najjar

**Affiliations:** 1 Internal Medicine, University of Florida College of Medicine, Jacksonville, USA; 2 Cardiology, University of Florida College of Medicine, Jacksonville, USA; 3 Pharmacology, University of Florida College of Medicine, Jacksonville, USA; 4 Pulmonary and Critical Care Medicine, University of Florida College of Medicine, Jacksonville, USA

**Keywords:** clinical pharmacology, ectopic cushing's syndrome, intensive and critical care, medical icu, small cell lung cancer (sclc)

## Abstract

A 67-year-old female presented with abdominal pain, nausea, vomiting, and unintentional weight loss. Further work-up revealed elevated serum cortisol, hypokalemia, and metabolic alkalosis in the setting of paraneoplastic Cushing's syndrome secondary to small lung cancer. The patient then developed refractory convulsive epileptic seizure despite being on multiple anti-epileptic medications. Here, we present a unique case where continuous etomidate infusion was used to lower serum cortisol, as adrenal insufficiency is associated with etomidate use. This case emphasizes how drug side effects can be used to achieve a desired treatment outcome.

## Introduction

Cushing's syndrome is a constellation of clinical findings that results from prolonged exposure to elevated levels of cortisol. Cushing's syndrome can be classified into two main subcategories. The first and the most common form results from supraphysiologic exposure to exogenous glucocorticoids, typically seen in patients being treated with long-term steroids for autoimmune or inflammatory disease [1]. Ectopic Cushing syndrome (EAS), which accounts for a remainder of endogenous causes, can be further subdivided by the origin of the adrenocorticotropin (ACTH) secreting tumor. Furthermore, of these cases, only approximately 1% to 5% are the result of small cell lung cancer (SCLC) [2]. The prognosis in this patient demographic is poor, with a mean survival of four to six months. To our knowledge, this is the first reported case of ectopic Cushing's syndrome in a patient with concomitant seizures secondary to hypercortisolism necessitating care in the intensive care unit (ICU). 

## Case presentation

**Figure 1:**
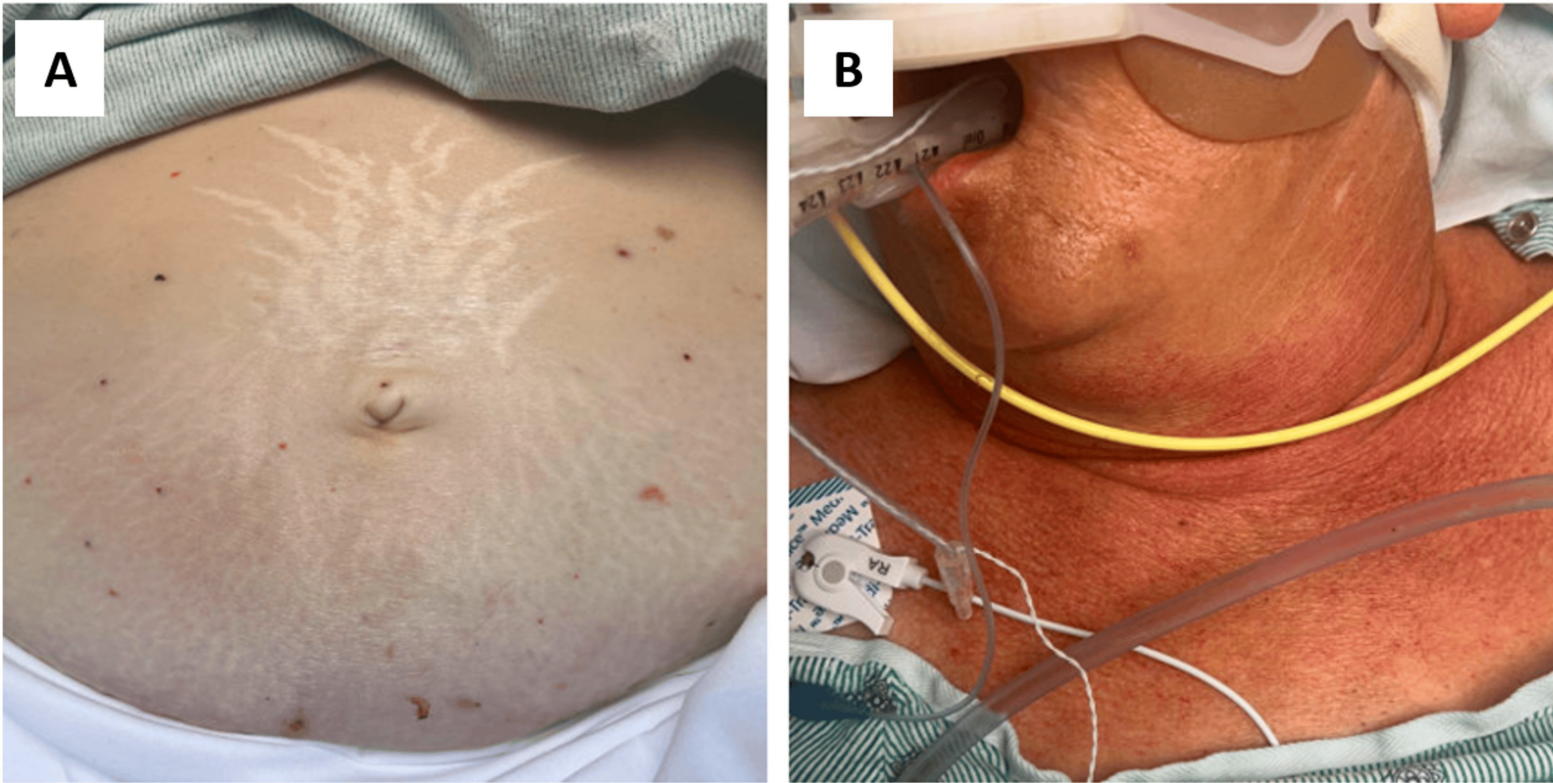
Physical exam findings demonstrating abdominal striae (A) and hyperpigmentation in sun-exposed areas (B)

A 65-year-old Caucasian female with a medical history pertinent for hypertension, hypothyroidism, uncontrolled type 2 diabetes mellitus, and 50-pack-year tobacco use presented to the emergency department complaining of abdominal pain, nausea, vomiting, and diarrhea for one day. The patient also admitted to orthopnea, worsening shortness of breath, bilateral lower extremity edema, and an unexplained 10-pound weight loss over several months. Physical exam was significant for moon-like facies, dorsocervical fat pad, hyperpigmentation, central obesity, wheezing, generalized abdominal tenderness with distention, and evidence of volume overload (Figure 1). Workup showed serum potassium of <1.5 mmol/L (reference range: 3.3 - 4.6), carbon dioxide of 43 mml/L (reference range: 21 - 29), chloride of 88mmol/L (reference range: 101 - 110), and platelet count of 95 x 10^3^/L (reference range: 140 - 440x10^3^). The patient was admitted to the medical ICU for further management of severe refractory hypokalemia requiring intravenous replacement.

**Figure 2:**
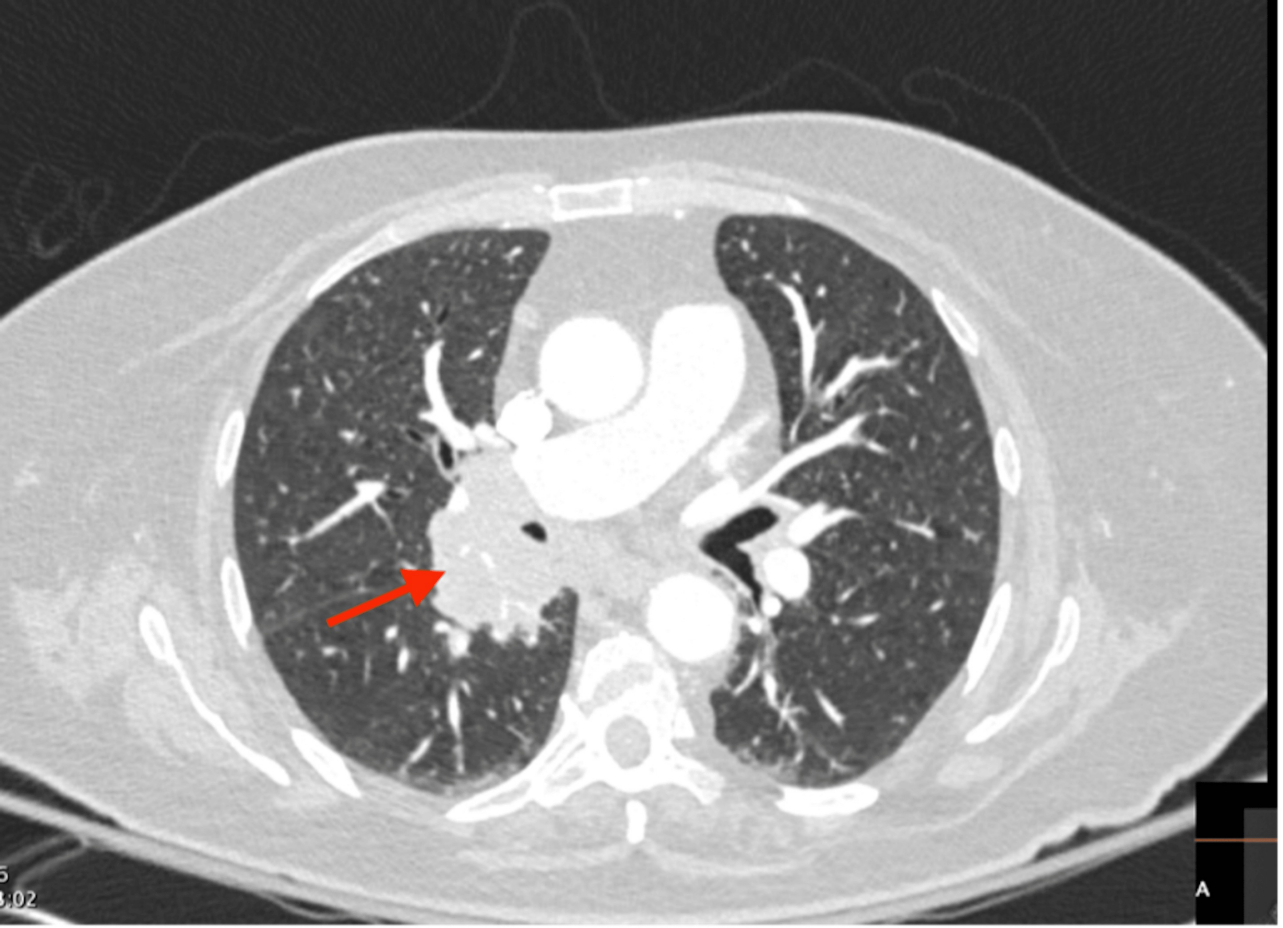
CT angiography of the chest showing a soft tissue mediastinal mass measuring 4.8 x 3.7 x 3.7 cm (red arrow)

**Figure 3:**
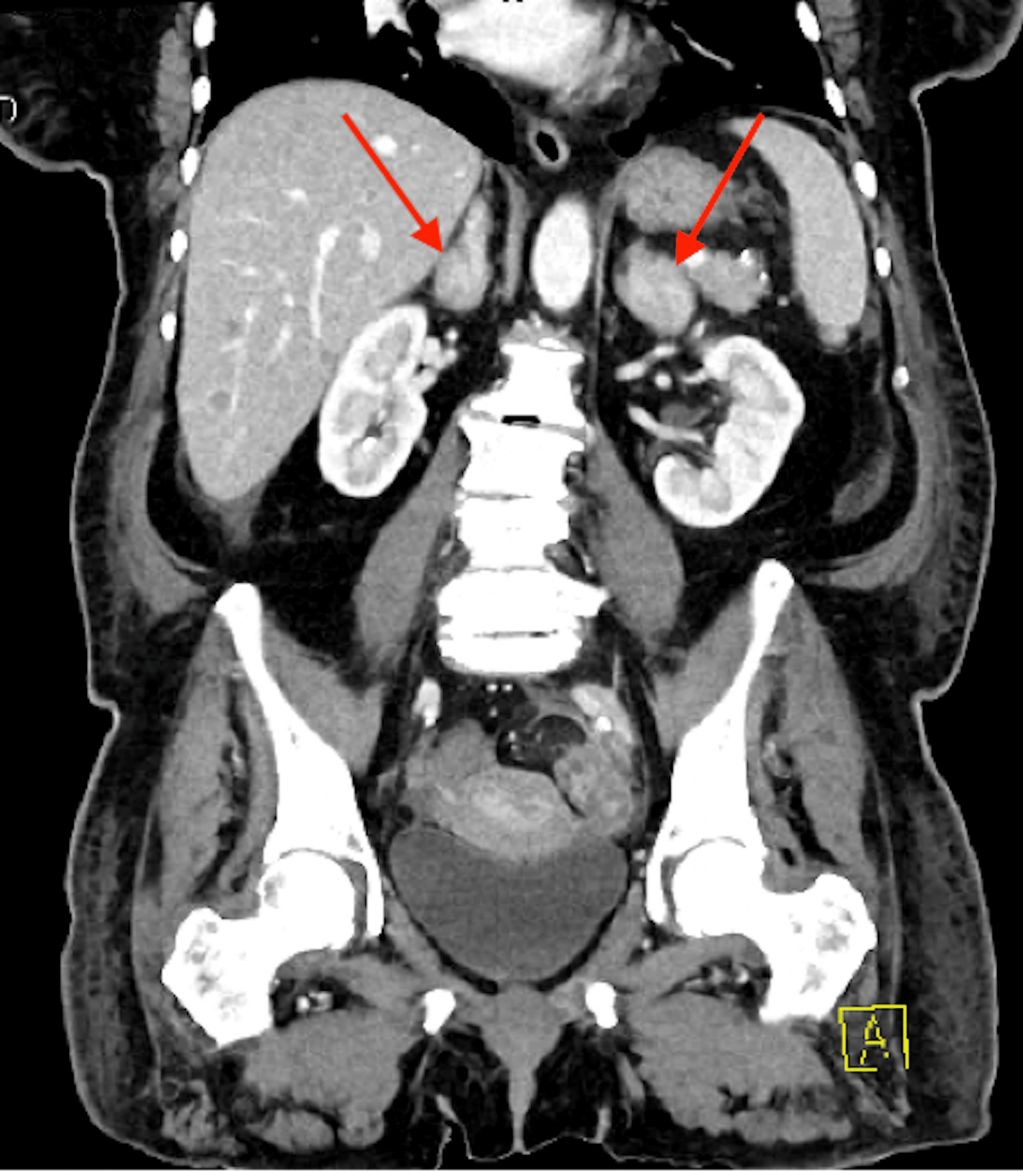
CT abdomen and pelvis with contrast displaying bilateral adrenal hyperplasia with multiple nodules (red arrows)

Initial imaging with computed tomography (CT) of the chest, abdomen, and pelvis demonstrated a 4.8 x 3.7 x 3.7 cm soft tissue mediastinal mass with lymphadenopathy and a 0.9 cm solid nodule in the right upper lung lobe (Figure 2). Additionally, there was bilateral adrenal hyperplasia with multiple nodules (Figure 3) all concerning for metastatic lung carcinoma.

Given the hypokalemia, metabolic alkalosis, hypertension, and elevated cortisol level along with physical exam and image findings, Cushing's syndrome was suspected in the setting of paraneoplastic syndrome. Further investigation showed an ACTH level of 134 pg/ml (reference range: 7.2 - 63.3) with an elevated cortisol level of 114 (reference range: 0.4 - 62.9). Later in the course, a high-dose dexamethasone test confirmed the diagnosis of ectopic ACTH secretion. An MRI of the brain and pituitary further ruled out the presence of a primary pituitary lesion.

On the second day of admission, the patient became comatose with hypertensive emergency. With a deteriorating clinical status, the patient had a tonic-clonic seizure and was ultimately intubated for airway protection. Continuous EEG indicated nonconvulsive status epilepticus and periodic lateralized epileptiform discharges (PLED) in the left temporal/occipital leads. At that time, the patient was initiated on midazolam and ketamine drip for burst suppression as recommended by the neurology team.

Bronchoscopy with endobronchial ultrasound (EBUS) was performed and confirmed the diagnosis of small cell lung cancer. Endocrinology was then consulted for suspected paraneoplastic ACTH. Due to the acuity of this patient's presentation, the endocrine team suggested an aggressive reduction in cortisol levels using an etomidate infusion. Intravenous etomidate drip was initiated at 0.02 mg/kg/hr with an up titration by 0.01 mg/kg/hr; a validated protocol by the Journalism of Endocrine Society [3-5].

On day eight of admission, the patient was at an etomidate dose of 8.6 mL/hr with cortisol levels measuring around 30. After further multidisciplinary discussion, a decision was made to start the patient on chemotherapy with carboplatin and etoposide in the hope of tumor eradication to minimize ectopic ACTH secretion. Once this therapy was started, the patient remained seizure free for the remainder of her hospital stay despite two episodes in which there were significant spikes in her cortisol level. 

After a prolonged hospital course with ventilatory support, persistent comatose state secondary to sedation and post-ictal state, hematological complications from chemotherapy, and multiple iatrogenic infections, the decision was made by the patient's healthcare proxy to terminate all aggressive treatment and proceeded with hospice for comfort care.

## Discussion

EAS secondary to small-cell lung cancer is a well-documented pathology that was first reported in 1962 by Liddle and colleagues [6]. Patients typically present with characteristic features of Cushing's syndrome however acute central nervous system involvement is rare. Given our patient's clinical picture and the absence of any history of seizures prior to hypercortisolemia, we posit that excessive endogenous steroid production was the culprit for our patient's seizures. It has been well documented that highly lipid-soluble steroids can easily cross the blood-brain barrier and influence neuronal function via binding to intracellular receptors. Glucocorticoids, for example, can alter certain neurochemical transmission processes, such as serotonin turnover or gamma-aminobutyric acid (GABA) uptake, subsequently causing an increase in seizure susceptibility [7]. The term 'neuroactive steroids' has been used to describe the interaction between steroids and neurotransmitter receptors [8]. Unfortunately, treatment options for seizures in this particular situation can be very challenging as there are no definitive guidelines. Etomidate, a sedative agent used for induction of general anesthesia, has shown promise in the acute management of EAS. Although literature on etomidate use in this demographic with seizures is sparse, several studies have demonstrated its effectiveness in the rapid correction of life-threatening complications of hypercortisolism [5,9].

Etomidate was initially developed in 1964 as an ultrashort-acting non-barbiturate induction anesthetic. One of its known side effects is its transient ability to inhibit the 11-betahydroxylase enzyme involved in steroidogenesis [9,10]. On this basis, the ICU team initiated a continuous etomidate infusion to lower cortisol levels in EAS despite its known seizure risk. After a detailed review of the presented patient's case, it is suggested that the protocol initially documented by Carroll et al. is an effective and safe way to manage severe hypercortisolism with acute CNS involvement [3].

**Figure 4:**
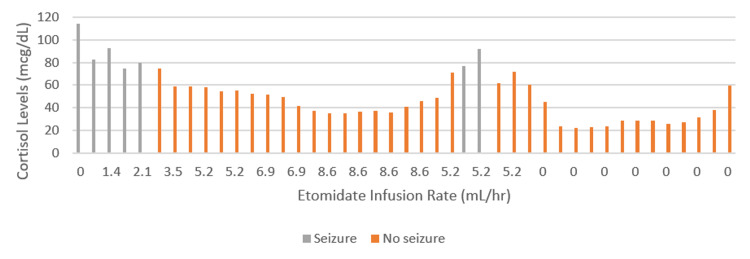
Bar graph demonstrating the relationship between the patient's increased cortisol levels and the incidence of seizure activity in the setting of a continuous etomidate infusion

The aforementioned statement is strongly supported by the data seen in Figure 4. The presented figure indicates that seizure activity was most abundant during the initiation phase of the etomidate drip. Moreover, these time points correspond with the highest recorded levels of cortisol. During this interval, the epileptologist reported that the patient had either tonic-clonic seizures, periodic lateralized epileptiform discharges (PLEDs), or continuous unconscious seizures (status epilepticus).

In order to reach a therapeutic level of etomidate, a titration was initiated and a maximum infusion rate of 8.6 mL/hr was achieved. At this dose, the patient remained seizure-free, which is believed to be a result of the reduced cortisol levels in combination with antiepileptics. It can be seen in Figure 4 that once etomidate was titrated down to a rate of 5.2 mL/hr with a corresponding increase in cortisol level ranging from 75-95mcg/dl, the patient had recurrent seizure episodes. Shortly after this recorded seizure activity, an additional antiepileptic medication was added along with the initiation of chemotherapy. Following this, the patient did not experience any additional seizures during the remaining two weeks of her hospitalization. In the presented case, the initiation of etomidate infusion was an essential measure used to bridge the patient to definitive therapy for EAS. Despite concerns for seizure precipitation, we surmise after careful review of our data points that etomidate was not the cause of our patient's seizures. In this case, the epileptiform activity was most likely caused by the elevated serum cortisol levels. This conclusion is further supported by the previously mentioned mechanism of neuroactive steroids.

According to literature, surgical excision of the primary tumor and its metastases is the ideal curative treatment for ACTH-producing tumors [11,12]. However, cases of SCLC can be challenging as a majority of patients have distant metastatic disease or bulky nodal disease. Typically, in cases where the paraneoplastic tumor is unresectable, the next best treatment is bilateral adrenalectomy (BLA). However, this treatment has only been suggested in salvage cases. Furthermore, when choosing a definitive treatment, it was decided that our patient was not a surgical candidate due to the acuity of her disease. After much consideration and with the family's consent, the oncology team recommended starting chemotherapy to treat the patient's SCLC. This case is unique as it highlights the use of a minimally studied etomidate infusion for the treatment of hypercortisolism in a patient with seizure activity. Etomidate is typically avoided in patients with a predisposition to seizures but in this case, the drug proved to be a safe and effective component of the medical team's armamentarium.

## Conclusions

This case emphasizes the importance of not only understanding the direct effects of medications but also recognizing how their side effects can be strategically utilized to achieve desired outcomes. Additionally, the complications encountered by our patient necessitated a multidisciplinary approach, which proved to be a vital aspect of her treatment. Each team involved provided crucial information, which resulted in the successful treatment of this patient's underlying condition. The open communication between the ICU team, endocrinology, neurology, and oncology is an aspect of this case that we strongly recommend being utilized when faced with similar clinical presentations.
